# Factors Associated with Impaired Humoral Immune Response to mRNA Vaccines in Patients with Inflammatory Bowel Disease: A Matched-Cohort Analysis from the RisCoin Study

**DOI:** 10.3390/vaccines13070673

**Published:** 2025-06-23

**Authors:** Katarina Csollarova, Leandra Koletzko, Thu Giang Le Thi, Paul R. Wratil, Ana Zhelyazkova, Simone Breiteneicher, Marcel Stern, Gaia Lupoli, Tobias Schwerd, Alexander Choukér, Veit Hornung, Oliver T. Keppler, Kristina Adorjan, Helga Paula Török, Sibylle Koletzko

**Affiliations:** 1 Department of Pediatrics, Dr. von Hauner Children’s Hospital, LMU University Hospital Munich, 80337 Munich, Germany; 2 Department of Medicine II, LMU University Hospital Munich, 81377 Munich, Germany; 3 Stiftung Kindergesundheit, c/o Dr. von Hauner Children’s Hospital, LMU University Hospital Munich, 80337 Munich, Germany; 4 Max von Pettenkofer Institute and Gene Center, Virology, National Reference Center for Retroviruses, LMU Munich, 80336 Munich, Germany; 5 German Center for Infection Research (DZIF), Partner Site Munich, 80336 Munich, Germany; 6 Institut für Notfallmedizin und Medizinmanagement (INM), LMU Klinikum, LMU Munich, 80336 Munich, Germany; 7 Laboratory of Translational Research Stress and Immunity, Department of Anesthesiology, LMU University Hospital, LMU Munich, 81377 Munich, Germany; 8 Gene Center and Department of Biochemistry, LMU Munich, 81377 Munich, Germany; hornung@genzentrum.lmu.de; 9 Department of Psychiatry and Psychotherapy, LMU University Hospital Munich, 80336 Munich, Germany; 10 University Hospital of Psychiatry and Psychotherapy, University of Bern, 3000 Bern, Switzerland; 11 Department of Pediatrics, Gastroenterology and Nutrition, School of Medicine Collegium Medicum, University of Warmia and Mazury, 10-561 Olsztyn, Poland

**Keywords:** SARS-CoV-2, anti-spike antibodies, virus neutralizing immunity, perceived stress questionnaire

## Abstract

**Background/Objectives:** The SARS-CoV-2 pandemic challenged patients with inflammatory bowel disease (IBD) under immunosuppressive therapies. We used data from the RisCoin cohort to investigate factors associated with a poor immune response to mRNA vaccination in these patients. **Methods**: From 4115 RisCoin participants, we matched 110 IBD patients by age and time interval since the second mRNA vaccination with 306 healthcare workers (HCW) without comorbidities (HCW-healthy) and 292 with medical conditions (HCW-plus); all were SARS-CoV-2 infection naïve. Basic questionnaires collected data on medication, COVID-19 vaccinations and side-effects, dietary patterns, lifestyle factors, and self-perceived stress. Main outcomes included anti-spike immunoglobulin levels and antibody-mediated live-virus neutralization immunity (NT) to the Omicron BA.1 variant (threshold NT ≥ 10 defined as IC50 values ≥1:10 serum dilution) after the second (baseline) and third vaccinations. **Results**: At baseline, IBD patients treated with anti-TNF but not those under vedolizumab or ustekinumab therapy had lower anti-spike levels compared to HCW-healthy and HCW-plus (166 versus 1384 and 1258 BAU/mL, respectively; *p* < 0.0001). Anti-TNF compared to vedolizumab/ustekinumab-treated patients reached NT titers above threshold in 17% versus 64%, respectively, and HCW-subgroups in 73% and 79% (all *p* < 0.0001). Current smokers showed a four to five times increased risk for non-neutralizing immunity compared to non-smokers. After the third vaccination, NT titers did not reach threshold in 15% anti-TNF compared to 5% vedolizumab/ustekinumab-treated patients and none of HCW (*p* < 0.01). Patients with IBD reported fewer clinical symptoms after vaccination. Perceived stress was not increased. **Conclusions**: Our findings support individualized schedules for mRNA-based vaccines in IBD patients with different immunosuppressive therapies and enforcement of non-smoking.

## 1. Introduction

The first year of the SARS-CoV-2 pandemic was a particular threat for patients with Crohn’s disease and ulcerative colitis, because most of them require long-term immune-suppressive therapy. Before the availability of COVID-19 vaccines, patients with inflammatory bowel diseases (IBD) and their families were very concerned about these therapies increasing their risk of acquiring infection and experiencing severe disease with increased mortality [1]. Data from large national and international registries and observational cohort studies provided convincing evidence that IBD patients are not more prone to acquiring SARS-CoV-2 infection compared to the general population [2,3]. However, these analyses did not consider that due to a higher self-estimated risk from COVID-19, patients under immunosuppression may take more precautions and preventive measures against infection, e.g., by avoiding crowding, social contacts, and the use of public transport [1]. Registry data identified IBD-specific risk factors that predispose patients to a severe disease course, as evidenced by the need for hospitalization, admission to an intensive care unit, and mortality. Only the use of systemic corticosteroids and a poorly controlled active bowel inflammation had been reported to increase the risk for complicated disease [4,5]. These findings resulted in the refinement of management recommendations and a pandemic-related update of the guidelines [3,6,7].

The availability of mRNA COVID-19 vaccines brought hope for a rapid and successful containment of the pandemic. However, concerns were raised about IBD-specific side effects, such as vaccination-related induction of disease flare and impaired effectiveness of the vaccines under mono- or combination therapy with different drugs that possibly affect the B-cell and T-cell-mediated immune response to vaccination. Assessing the effectiveness of COVID-19 vaccines in patients under immunosuppressive drugs is complex due to the influence of various factors. These include patient characteristics such as age, gender, genetic predisposition, comorbidities, and lifestyle choices and the specific type of COVID-19 vaccine administered. The interaction of different factors further complicates the analysis of single effects, even when confounders are considered. The RisCoin Study, a prospective longitudinal monocentric observational cohort study conducted at the LMU University Hospital, Munich, Germany, addressed this complexity [8]. In this manuscript, we focused on adult patients with IBD treated with various drugs, with particular interest in the biologics anti-TNF antibodies, vedolizumab, and ustekinumab. TNF is a key cytokine involved in the activation and differentiation of immune cells, playing an essential role in T-cell-dependent antibody production—such as the activation of circulating T follicular helper cells—and in the formation of germinal centers, where B cells mature, diversify, and develop into antibody-producing memory B cells and long-lived plasma cells. In contrast, ustekinumab specifically targets the p40 subunit shared by IL-12 and IL-23, thereby blocking their signaling but not interfering with the overall function of T or B cells involved in vaccine responses. Similarly, vedolizumab acts by inhibiting the trafficking of T cells to the gut and does not impact the immune response to systemically administered vaccines.

We investigated humoral immune responses to mRNA vaccination in IBD patients after basic and booster immunization and compared them to those in health care workers (HCW) without and those with underlying diseases. Furthermore, we assessed epidemiological and environmental factors potentially affecting the observed immune responses.

## 2. Materials and Methods

### 2.1. RisCoin Study

Detailed information on the RisCoin study has been previously published [8]. In brief, all participants were enrolled at the LMU University Hospital Munich between 7 October and 16 December 2021, during a COVID-19 booster vaccination campaign organized by the hospital. Inclusion criteria for the participation were as follows: (i) completed basic SARS-CoV-2 immunization (at least two vaccinations) with the last vaccine ≥4 weeks prior to enrollment; (ii) age ≥18 years; and (iii) signed informed consent. At enrollment, participants donated blood samples for assessment of SARS-CoV-2-specific antibody concentrations and antibody-mediated live-virus neutralization. Participants completed an extensive questionnaire covering demographics, their living and occupational situation, details on adverse effects of COVID-19 vaccinations, previous influenza immunizations, pre-existing health conditions and allergies, regular intake of medication, vitamins, and supplements, dietary habits, and lifestyle factors including tobacco and alcohol consumption [8].

To evaluate mental stress, we utilized the validated German short version of the standardized Perceived Stress Questionnaire (PSQ) [9,10]. This questionnaire provides an overall stress score as well as scores across four domains: (i) worries/fears about the future and feelings of despair and frustration, (ii) tension encompassing restlessness, fatigue, and lack of relaxing, (iii) joy highlighting positive aspects such as feeling challenged, motivated, and secure, and (iv) demands measuring time constraints, pressure, and feeling overwhelmed.

Throughout the entire study period, participants were asked to complete short questionnaires weekly or event-related via a study app. These short questionnaires inquired about information regarding common clinical symptoms of COVID-19, COVID-19 test results, and the occurrence of any SARS-CoV-2 breakthrough infection confirmed by a PCR test. Further, participants could report details regarding their COVID-19 booster vaccination(s) including adverse effects and related countermeasures such as intake of antipyretics [8,11].

Follow-up visits took place from 13 December 2021 to 15 March 2022 and, subsequently, from 19 September 2022 to 6 October 2022 [8]. Participants were invited to additional SARS-CoV-2 antibody testing if they (i) received a booster vaccine after enrollment and ≥4 weeks prior to the follow-up visit or (ii) after they reported a PCR-confirmed breakthrough infection, a suspected infection with typical clinical symptoms, a positive rapid antigen test, or experiencing a close contact with a SARS-CoV-2 PCR-positive person.

### 2.2. Serological Vaccine Immune Response

The humoral immune response to SARS-CoV-2 was determined by measuring anti-spike and anti-nucleocapsid antibodies via the Elecsys Anti-SARS-CoV-2 N (Roche, Basel, Switzerland, cat.: 09203095190) and Elecsys Anti-SARS-CoV-2 S (Roche, Basel, Switzerland, cat.: 09289267190), respectively, which were performed in accordance with the manufacturer’s recommendations [11]. Anti-SARS-CoV-2 spike antibody levels were quantified as binding antibody units per milliliter (BAU/mL).

To assess antibody-mediated live-virus neutralization, a highly predictive biomarker of immune protection from symptomatic SARS-CoV-2 infection and severe COVID-19 [12,13], a live-virus neutralization assay was employed as described with a clinical isolate of SARS-CoV-2 variant Omicron B.1.1.529 BA.1 (GISAID EPI ISL: 7808190) [12,14]. Neutralization titers were calculated as the half-maximal effective dilution of each serum to neutralize viral infection. The upper threshold for the detection of neutralizing activity in the participants’ sera was set to ≥1:10 serum dilution.

### 2.3. Data Extraction

For this analysis, we extracted data from participants with IBD who were receiving regular care in one of the IBD-Clinics at the LMU University Hospital Munich and from participating HCW. Only data that were complete regarding age, gender, type or date of all previous COVID-19 vaccinations, and key covariates at enrollment (baseline) and at the first follow-up (FU) were extracted (Supplementary File S1). We excluded participants who had already received their third COVID-19 vaccination (booster) at enrollment, those with a PCR-confirmed SARS-CoV-2 infection prior to or within 2 weeks of recruitment, and those with reactive anti-SARS-CoV-2 nucleocapsid antibodies indicating a subacute or resolved COVID-19. Additionally, we excluded participants who had received non-mRNA vaccines (Supplementary File S1).

#### 2.3.1. IBD Cohort

We included adult patients with Crohn’s disease or ulcerative colitis. These individuals completed an additional questionnaire on their current IBD-specific medication, which we categorized into three different groups: (i) anti-TNF antibodies (infliximab, adalimumab or golimumab), (ii) no anti-TNF, but vedolizumab or ustekinumab, and (iii) other IBD-specific medications than those in groups i and ii. For group ii, we first investigated the immune response to basic COVID-19 vaccination, stratified by treatment with either vedolizumab or ustekinumab. Since no significant difference or even a trend was found with respect to the vaccination immune response, we combined patients treated with vedolizumab or ustekinumab in one group (group ii).

#### 2.3.2. HCW Cohorts as Comparator

The HCW cohort served as a control group for the IBD cohort, as they may experience similar environmental exposures as patients but are generally healthier. Based on comorbidities reported in the initial questionnaire and current use of any medication, we divided the HCW cohort into three subgroups: HCW-healthy, HCW-plus, and HCW with current immunosuppressive therapy [8]. The rationale for stratification of the HCW cohort was also provided in the details in Supplementary File S2. HCW-healthy reported no current or regular intake of any medication (excluding vitamins and supplements) as well as the absence of the following diseases: chronic cardiovascular, pulmonary, renal, gastro-intestinal, liver, neurological, hematological, rheumatological or (auto)immune disease, diabetes mellitus, dyslipidemia or other metabolic disorders, thyroid or other endocrinological disease, and current or previous malignancy. HCW with overweight or obesity, those reporting food or contact allergies, and those with allergic rhino conjunctivitis without current drug therapy were considered healthy. In contrast, HCW-plus was defined as HCW reporting any of the above-mentioned co-morbidities or regular intake of medication excluding immunosuppressive drugs. HCW currently treated with immunosuppressive drugs were not included as controls due to the limited number of participants and the heterogeneity regarding immunosuppressive treatment in this subgroup (Supplementary Files S1 and S2).

### 2.4. Statistical Analysis

#### 2.4.1. Stratification Matching

At the exploration step, our data revealed that age, but not gender, and time since the second COVID-19 vaccination were significantly associated with the humoral immune response as quantified by anti-spike IgG (BAU/mL) and neutralization antibody titers (NT) (Supplementary File S2). To mitigate the confounding effect of age and the time interval since the latest vaccination on the immune response, we applied stratification matching. Herein, randomly selected participants from HCW-healthy and HCW-plus within each age category (18–30, 31–40, 41–50, 51–60, >60 years) and time interval (>6 months or ≤6 months) since the date of sample collection to the second vaccination were matched with individuals from the IBD cohort. We strived to achieve the highest possible matching ratio. Details of the stratification matching for the IBD cohort are provided in Supplementary File S2.

#### 2.4.2. Descriptive Statistics

Descriptive statistics were presented using median and interquartile range (IQR) from the 25th to the 75th percentile for continuous variables, while frequency (n) and percentage (%) were used for categorical variables. To determine the significant difference in the proportion of respective factors between the IBD cohort and its matched HCW cohorts, the Mann–Whitney U-test was employed for continuous variables, and Pearson’s Chi-square test was used for categorical variables.

When comparing quantitative anti-spike Ig in IBD cohort and its matched HCW cohorts or in different strata among IBD patients, we conducted the Kruskal–Wallis test and afterwards applied the Dwass, Steel, and Critchlow-Fligner method to assess pairwise two-sided multiple comparisons analysis of quantitative anti-spike levels between different strata while controlling for the overall error level.

#### 2.4.3. Multivariable Analysis

To investigate factors associated with quantitative anti-spike Ig among IBD patients, we performed a multivariable Generalized Linear Model (GLM) applying log-nature transformation of anti-spike Ig adjusted for gamma distribution due to the positive-skewed non-normal distribution of anti-spike Ig. All factors associated with a higher quantitative anti-spike Ig (*p*-value ≤ 0.25) were included in the multivariable GLM. After applying backward elimination and adjusting for age (years), gender, IBD type, and time interval from second vaccination to enrollment, the final multivariable GLM was achieved with no missing co-variates. Results are given as coefficients with their 95% confidence interval (CI) and *p*-value determining the significance of their respective coefficients obtained from the Wald chi-square test.

Neutralization activities of participants were categorized as non-neutralizing if their neutralization titers (NT) were above the threshold (≥1:10 serum dilution) and as neutralizing if their NT was <1:10 serum dilution. Pearson’s chi-square test was performed to determine the significant difference in the proportion of non-neutralizing NT and neutralizing NT across different strata among IBD patients as well as comparing the IBD cohort with its matched cohorts. In the case of multiple comparisons, Holm–Bonferroni correction for *p*-value was applied.

To identify the factors associated with non-neutralizing NT among patients with IBD, we performed a multivariable logistic regression. All variables that showed a higher likelihood of non-neutralizing NT (*p*-value ≤ 0.25) were included in the analysis. The final model was determined after backward elimination and adjusted for gender, age (in years), type of IBD, co-existing underlying diseases, and the time difference (in months) between the second vaccination and enrollment. The odds ratios (OR) and 95% confidence intervals (CI) with their corresponding *p*-values from the Wald chi-square test are reported.

All statistical tests were conducted with a two-sided significance level of 5%. Statistical analyses were performed using SAS Enterprise Guide 8.1 (Statistical Analysis Software, SAS Institute Inc., Cary, NC, USA) and Prism 10.2.1 (GraphPad Software, Boston, MA, USA).

## 3. Results

### 3.1. Characteristics of the Study Population

Out of 4115 participants with serology results in the RisCoin study [8], inclusion criteria for the immune response analysis and stratification matching were met for 110 of 180 enrolled patients with IBD, for 1512 HCW-healthy, as well as 1262 HCW-plus with underlying diseases and no current immunosuppressive therapy (Supplementary Files S1 and S2). The 110 IBD patients were matched by age groups and time since the second COVID-19 vaccination to HCW-healthy (n = 306) and HCW-plus (n = 292). Comparison of the cohorts before and after matching is shown in Supplementary File S2. Almost all IBD patients received advanced therapies, most of them biologics: anti-TNF antibodies (n = 53), ustekinumab, or/and vedolizumab (n = 44). The third subgroup of individuals without biologics treatment consisted of 13 patients treated with kinase inhibitors (tofacitinib, n = 2), with immunomodulators (azathioprine or tacrolimus, n = 5), with prednisolone (less than 20 mg/d, n = 2), and with mesalazine only (n = 4). Given the small number of patients without biologics, these patients were combined into one subgroup and analyzed separately.

The baseline characteristics of the IBD cohort compared to matched HCW-healthy and HCW-plus are shown in Table 1. There were more females in the HCW-healthy (66%) and HCW-plus (82%) groups compared to the IBD cohort (45%). The median BMI of patients with IBD was higher than that of HCW-healthy individuals, with significant differences in the distribution of BMI categories between the two groups. The frequency and intensity of clinical symptoms after the second COVID-19 vaccination were significantly lower in patients with IBD than in both HCW subgroups. More IBD patients than HCW had received influenza vaccination during the previous winter season (2020/2021). We found significant differences between the IBD and the two HCW-groups regarding regular intake of food supplements, e.g., vitamin D, multivitamins, minerals, trace elements, and fish oil. The percentage of active smokers was significantly higher in IBD patients (20%) than in HCW-healthy (12%) but comparable to HCW-plus participants (17%) (Table 1).

Further comparison of the matched subgroups regarding different lifestyle factors is given in Supplementary File S3. IBD patients reported any or regular alcohol consumption less often compared to matched HCW-healthy and HCW-plus participants. With respect to dietary habits, IBD patients reported more frequent consumption of meat compared to the two HCW subgroups, but they consumed fewer portions of vegetables and fruits per day. Twenty percent of IBD patients reported avoiding special foods due to food allergy or intolerance compared to only 4% of HCW-healthy and 8% of HCW-plus participants, both *p* < 0.001. Compared to HCW-healthy, significantly fewer patients with IBD reported traveling abroad in the 12 months prior to enrollment. No differences were observed between the groups regarding the frequency of moderate to heavy physical activity per week.

### 3.2. Comparison of Anti-Spike Ig Titers After Basic Immunization

**Figure 1:**
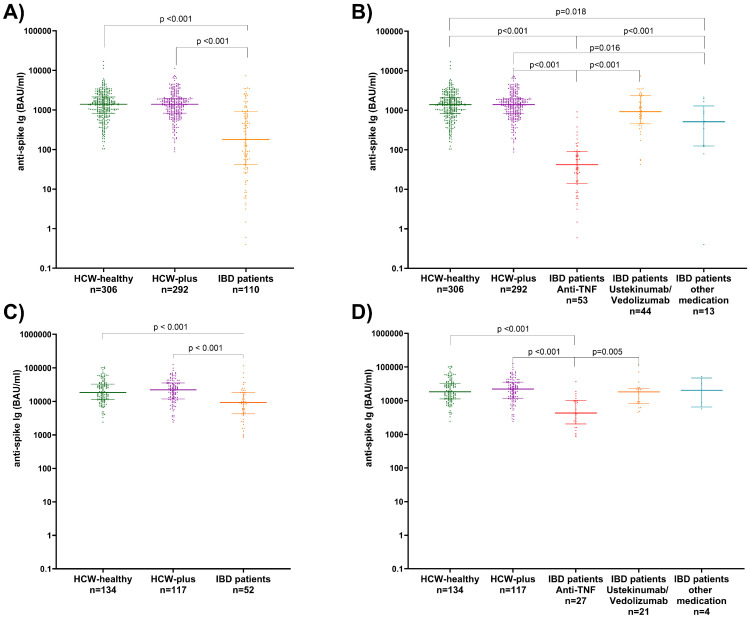
Quantitative anti-spike antibody titers in adult patients with IBD as total cohort (**A**,**C**) and after stratification by type of IBD medication (**B**,**D**), healthy HCW (HCW-healthy) and HCW with underlying diseases (HCW-plus) at baseline (**A**,**B**) and follow-up (**C**,**D**).

The quantitative titers of anti-spike Ig antibodies after two COVID-19 mRNA vaccinations in IBD patients showed a larger range with a significantly lower median value compared to the matched HCW-healthy (166 vs. 1384 BAU/mL, *p* < 0.001) and HCW-plus subgroup (166 vs. 1258 BAU/mL, *p* < 0.001), with no significant difference between the two HCW subgroups regarding anti-spike Ig titers (Figure 1A). When IBD patients were stratified by medication, only IBD patients treated with anti-TNF antibodies had significantly lower anti-spike Ig titers compared to both HCW groups (both *p* < 0.001) and also compared to IBD patients treated with ustekinumab/vedolizumab (*p* < 0.001) as well as IBD patients with other medications (*p* < 0.001) (Figure 1B). IBD patients treated with ustekinumab or vedolizumab had anti-spike Ig titers that were comparable to the two HCW subgroups. Patients of the third IBD subgroup (i.e., other medications) had slightly lower antibody titers compared to HCW healthy (*p* = 0.018) and HCW-plus (*p* = 0.016) (Figure 1B).

### 3.3. Factors Associated with Anti-Spike Ig Titers After Basic Immunization in Patients with IBD

We used multivariable generalized linear modeling to identify factors associated with anti-spike-Ig titers after basic immunization in adult IBD patients. Compared to treatment with ustekinumab or vedolizumab, treatment with anti-TNF antibodies was confirmed as the most significant factor associated with lower anti-spike antibody concentrations (OR 0.04; 95% CI: 0.03–0.06, *p* < 0.001) (Table 2). Other factors associated with lower anti-spike Ig level included female versus male gender (*p* = 0.004), having chronic pulmonary disease (*p* = 0.001), and active smoking behavior (*p* = 0.010). The subtype of IBD and the time since the second vaccination showed no significant impact on Ig titers (Table 2).

### 3.4. Comparison of Virus Neutralization Activities After Basic Immunization

**Figure 2:**
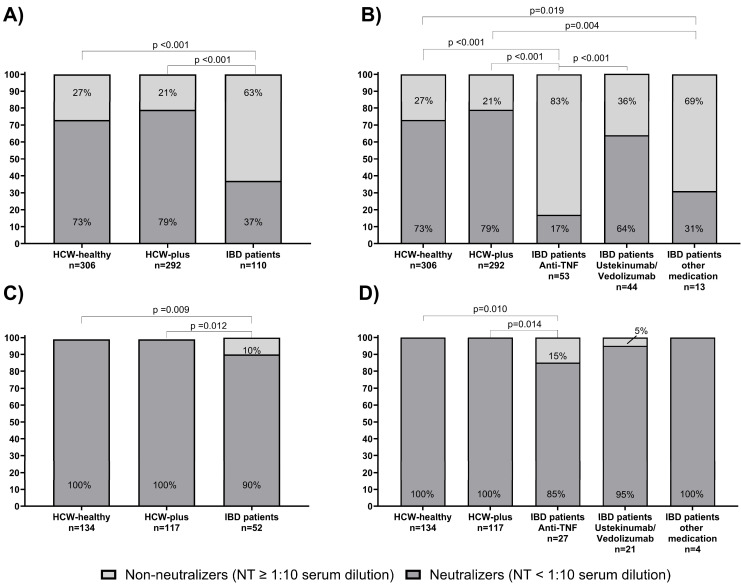
Frequencies of non-neutralizing (NT ≥ 1:10 serum dilution) and neutralizing (<1:10 serum dilution) antibody titers among adult patients with IBD as total cohort (**A**,**C**) and after stratification by type of IBD-medication (**B**,**D**) compared to healthy HCW (HCW-healthy) and HCW with underlying diseases (HCW-plus) at baseline (**A**,**B**) and follow-up (**C**,**D**).

The rates of participants with no neutralization activity against SARS-CoV-2 Omicron BA.1 and of confirmed neutralizers after the second vaccination in the three matched groups are depicted in Figure 2A. Only 37% of IBD patients showed Omicron BA.1-specific neutralization activity compared to 73% of HCW-healthy and 79% of HCW-plus (both *p* < 0.001). After stratification of the IBD cohort into subgroups according to their medication, the lowest proportion of patients having neutralization activity against the virus was the anti-TNF group (17%). Patients with ustekinumab or vedolizumab therapy, in comparison, had 64% neutralizers, and patients with other IBD drugs had 31% (Figure 2B).

### 3.5. Factors Associated with Insufficient Neutralizing Activities Against SARS-CoV-2 Omicron BA.1 After Basic Immunization in Patients with IBD

When we compared non-neutralizers among the IBD patients with those that showed neutralizing activity against Omicron BA.1, we found no significant relation to gender, age group, BMI, type of IBD, additional morbidity, symptoms after the first or second vaccination, or most assessed lifestyle factors (Table 3 and Supplementary File S4). The only significant results in the univariate analysis were the type of IBD medication and smoking behavior.

**Figure 3:**
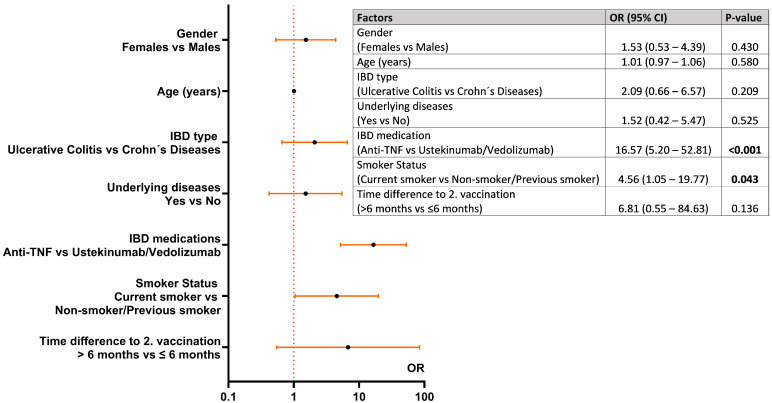
Factors associated with non-neutralizing antibody titers (NT ≥ 1:10 serum dilution) measured after second COVID-19 vaccination in adult patients with IBD using multivariable logistic regression.

In the multivariable logistic regression analysis (Figure 3), these two factors were confirmed to be significantly associated with having non-neutralizing activity against the virus. Patients treated with anti-TNF antibodies had a 16 times higher risk for being non-neutralizers than patients with IBD receiving ustekinumab or vedolizumab treatment (*p* < 0.001). Active smokers had a four to five times higher risk than non-smokers or previous smokers (*p* = 0.043) (Figure 3). Age, gender, IBD type, and having another co-morbidity had no impact on the measured NT.

### 3.6. Follow-Up After the Third Vaccination (Booster)

Blood sampling at least 4 weeks after the third COVID-19 vaccination was performed in 1784 participants (1694 HCW and 90 patients with IBD). After applying the same exclusion criteria as for the initial case-control analysis, 52 patients with IBD remained eligible for analysis. These were matched to 134 HCW-healthy and 117 HCW-plus.

### 3.7. Comparison of Anti-Spike Ig Concentrations After Booster Vaccination

After the third vaccination, a significant difference in median anti-spike Ig titers remained between patients with IBD (9328 BAU/mL), HCW-healthy (18,553 BAU/mL), and HCW-plus (22,350 BAU/mL), both *p* < 0.001 (Figure 1C). When stratified by medication, only anti-TNF-treated IBD patients had significantly lower anti-spike titers compared to the other subgroups (Figure 1D).

**Figure 4:**
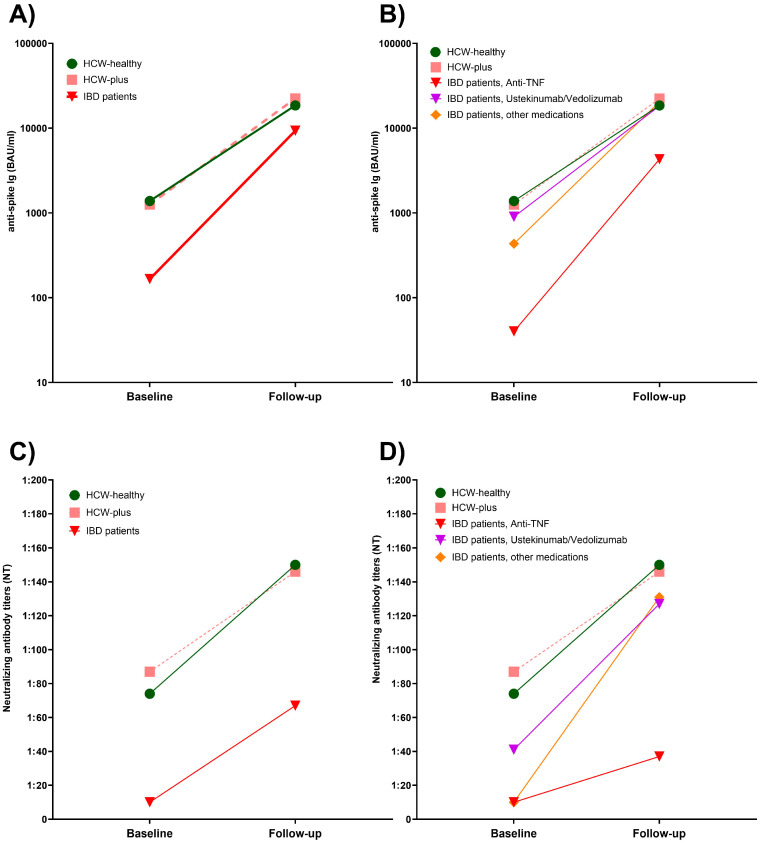
Booster effect after the third vaccination on anti-spike levels (**A**,**B**) and neutralization titers (NT) (**C**,**D**) among adult IBD patients in total (**A**,**C**) and after stratification by type of IBD-medication (**B**,**D**) as well as in healthy HCW (HCW-healthy) and HCW with underlying diseases (HCW-plus).

Figure 4A depicts the median titers of the three matched groups at baseline and after the booster vaccination. As shown here, there was no significant difference in the median anti-SARS-CoV-2 spike levels in HCW-healthy compared to HCW-plus. Although patients with IBD showed a strong increase in antibody concentrations after the booster vaccination, their median titers did not reach levels that were comparable to those of the HCW groups. Figure 4B demonstrates that an impaired antibody response to booster vaccination was only observed in the subgroup of patients with IBD receiving anti-TNF antibody therapy. After booster vaccination, IBD patients treated with vedolizumab or ustekinumab or other IBD medications reached anti-spike titers that were similar to those in the matched HCW groups.

### 3.8. Factors Associated with Anti-Spike IgG Titers After the Third Vaccination

Multivariable regression analysis (GLM) confirmed that anti-TNF antibody therapy was a significant risk factor for low anti-spike titers compared to ustekinumab/vedolizumab therapy (*p* < 0.001) (Table 2). In contrast to the analysis after basic immunization, the type of IBD was significantly related to anti-spike titers after adjusting for other possible confounders, with patients with ulcerative colitis having higher titers than those with Crohn’s disease (*p* = 0.007).

### 3.9. Comparison of SARS-CoV-2 Omicron BA.1 Neutralizing Activities After the Third Vaccination

After booster vaccination, all HCW and 90% of IBD patients (85% with anti-TNF therapies, 95% with ustekinumab/vedolizumab, and 100% with other IBD medications) showed neutralizing activity against Omicron BA.1 (Figure 2C,D). Significant differences in the proportion of individuals with neutralizing activity were found only between patients with IBD receiving anti-TNF therapies and the two HCW groups (*p* = 0.010 and *p* = 0.014, respectively).

The comparison of neutralization titers at baseline and after booster vaccination in the three matched groups is given in Figure 4C. Although titers were significantly increased, the median titers after the third vaccinations in IBD patients remained lower than those in the two HCW groups after baseline immunization. Figure 4D depicts the marked differences according to the IBD medications used. Patients treated with anti-TNF antibodies reached comparably low titers after booster vaccination.

### 3.10. Perceived Stress Questionnaire Score (PSQ-Score)

The results for the mean total PSQ-Score and for the four specific subdomains in the three matched groups at enrollment are shown in the Supplementary File S5A–C. The mean total PSQ-Scores in HCW-healthy was 33, which is equal to the mean values found in 334 healthy adults of similar age in a previous publication from Germany [10]. The mean total score in HCW-plus was significantly higher compared to the HCW-healthy group (43 vs. 33, *p* < 0.001) (Supplementary File S5A). The percentage of HCW-plus individuals with values above 33, indicating increased level of stress, was higher than in HCW-healthy (63% vs. 48%, *p* < 0.001) (Supplementary File S5B). IBD patients showed for the total score and the domains “tension,” “worries,” and “demands” similar values to HCW-healthy individuals but lower scores compared to HCW-plus individuals (*p* < 0.018). Values in the domain “joy” were significantly lower in IBD patients compared to HCW-healthy (*p* < 0.015) and comparable to those in the HCW-plus group (Supplementary File S5C).

## 4. Discussion

This study explored the risk factors associated with impaired humoral immune responses to COVID-19 mRNA vaccines in SARS-CoV-2 infection-naïve IBD patients, utilizing data from the RisCoin study [8]. Beyond medical conditions and medication, we examined the influence of a wide range of demographic and lifestyle factors [8]. The large number of HCW included in the original study (see Supplementary File S1) enabled us to create two well-matched control groups (matched in a 1:3 ratio): one consisting of healthy HCW and the other comprising HCW with underlying medical conditions. This approach effectively mitigated the confounding effects of age and temporal distance to the latest vaccination on humoral immune responses [15].

Our findings reveal that IBD patients under immunosuppressive medication with anti-TNF antibodies exhibit significantly lower median anti-spike concentrations after basic immunization compared to both HCW control groups and to IBD patients treated with ustekinumab/vedolizumab. The differential effect of these therapies on vaccine-induced humoral immunity is consistent with previous publications [16,17,18]. Neutralizing antibody titers, a key correlate of protection, were similarly affected with only 17% of IBD patients on anti-TNF therapies achieving neutralizing activities against SARS-CoV-2 Omicron BA.1 above threshold after basic immunization, compared to over 70% in both HCW groups and 65% in patients on ustekinumab/vedolizumab therapies. Multivariable regression analysis confirmed that anti-TNF therapy was the strongest negative predictor of anti-spike levels and neutralization titers, which is in line with recent literature [16,19].

The booster vaccination significantly improved antibody responses across all groups, but IBD patients on anti-TNF therapy continued to show significantly lower median anti-spike antibody levels and neutralization activities compared to HCW, with still 15% of them being non-neutralizers. Notably, under anti-TNF-therapies, the median NT after booster remained lower than titers in HCW after basic immunization (Figure 4D). Our results are in accordance with existing data on the efficacy of booster vaccination in patients with impaired humoral responses due to anti-TNF therapies [16,18,20,21]. Taken together, these data combined with the previously observed accelerated decline of antibody titers under anti-TNF therapies [17] sustain an individualized vaccination schedule with (repeated) booster vaccination to improve and extend immune responses in IBD patients receiving anti-TNF therapies. In contrast, patients receiving ustekinumab or vedolizumab achieved antibody levels comparable to HCWs, suggesting that mRNA vaccine booster doses may effectively overcome some of the immunosuppressive effects associated with these treatments. Due to the small number of IBD patients treated with other drugs in our cohort, e.g., tofacitinib and immunomodulators, our study does not allow for any conclusions regarding their association with the humoral immune response.

The marked impairment of SARS-CoV-2 neutralization activities among anti-TNF-treated IBD patients is a critical finding, as these could potentially lead to dismal protection from symptomatic SARS-CoV-2 infection and severe COVID-19 [12,13]. Reduced neutralization capacities may contribute to reduced viral clearance and longer-term infections, which may favor virus adaptation to the host adaptation and the evolution of novel virus variants [22]. Repeated booster vaccination with variant adapted vaccines could be beneficial for patients treated with anti-TNF, and mucosal vaccination strategies should emerge [16,21].

Besides medication, our study evaluated the impact on humoral vaccine response of a wide variety of demographic factors, dietary habits, regular intake of vitamins and supplements, lifestyle factors including tobacco and alcohol consumption, and co-morbidities. Among the investigated factors, female gender, chronic pulmonary disease, and active smoking status were independently associated with lower anti-spike antibody concentrations after basic immunization, while smoking status was associated with showing no neutralization activity. After booster vaccination, all these factors lost their significant impact, but the phenotype of Crohn’s disease was associated with lower antibody titers compared to ulcerative colitis. Although in line with another publication [19], this finding should be interpreted with caution, given the small number of participants at follow-up.

Besides the known male bias for COVID-19 severity and mortality [23], sex differences have also been described for immunogenicity, with females having higher antibody titers than males after conventional [24] and mRNA vaccinations [25]. In our IBD cohort, female gender was associated with lower spike antibody titers after basic immunization, which may have resulted in a selection bias.

Having a chronic pulmonary disease and active smoking behavior were independent risk factors for lower anti-spike antibody titers after basic immunization in patients with IBD. Current smoking was associated with a four to five times higher risk for being a non-neutralizer after basic immunization. Twenty percent of our IBD patients were current smokers, despite the known negative effects of smoking on the course of IBD [26]. This percentage was higher compared to the 8–9% reported in an IBD cohort from UK [19]. The observation of an impaired humoral immune response to mRNA vaccination in active smokers is in line with data in patients with IBD and various other disease entities and healthy control populations [20,27,28], confirmed by meta-analyses [29]. An individualized vaccine schedule should also consider active smoking in patients with IBD. These individuals are also prone to a more severe disease course, increased rates of hospitalization and surgery, and the need for more aggressive immunosuppressive therapies [30].

Our study design allowed us to perform a comprehensive dietary, lifestyle, and perceived stress analysis in patients with IBD compared to HCW during the COVID-19 pandemic. As expected, we found significant differences between groups regarding regular intake of vitamin D supplements, reflecting recommendations given in our IBD clinic. A higher proportion of patients with IBD reported avoidance of special foods and consuming fewer portions of vegetables and fruits per day, which, although not advisable, is an expected finding [31]. Complete avoidance of alcohol was more often reported in IBD patients compared to controls, although data about its harmful effect in IBD are less solid than for smoking. The higher median BMI observed in the IBD cohort is most likely multifactorial (e.g., systemic inflammation, steroid use, or reduced physical activity) [32], but like smoking, this calls for more attention and counseling in clinical care. The reported avoidance of traveling aboard among IBD patients may be related to a more precautious life style due to a higher self-estimated risk during the pandemic [1].

Our patients with IBD performed well in the PSQ, obtaining comparable values with healthy HCW for the domains “tension,” “worries,” and “demands” and lower scores compared to HCW with associated medical conditions (HCW-plus). This may partly be attributed to our regular newsletter updates on scientific findings and recommendations for patients with IBD during the pandemic [1]. The self-perceived stress level was lower compared to that of 144 IBD patients enrolled in the validation study of the German short version of PSQ [10]. However, that study was performed 20 years ago, before the availability of most current IBD drugs with a more favorable benefit-to-harm ratio.

The reduced frequency and intensity of clinical symptoms after the second COVID-19 vaccination in IBD patients compared to HCW groups indicate the blunted immune response in this population, potentially due to immunosuppressive therapies [33]. The good tolerability of the vaccine and the fact that IBD patients already had higher rates of influenza vaccination predict a good acceptance of COVID-19 vaccination in the IBD population. Indeed, current observations confirm an overall positive attitude toward COVID-19 vaccination in IBD patients, which is an encouraging finding [34].

Our study has several strengths. The use of well-matched control groups and stratification based on age and vaccination timing reduced potential confounding effects. Only participants who received a homologous mRNA vaccine regimen for primary immunization and booster doses were included. Additionally, individuals with known prior SARS-CoV-2 infection or positive nucleocapsid antibodies were excluded. This eliminated the effects of heterologous vaccination or hybrid immunity, both of which have been shown to impact vaccine response [35,36,37]. Limitations include female dominance in the HCW cohort, which did not allow a 3:1 matching with the IBD patients, and a high loss rate of approximately 50% of our cohort until the follow-up. This was in part due to exclusions of participants who had COVID-19 between the baseline and the follow-up time points.

## 5. Conclusions

In conclusion, our analysis, conducted in a homogenous and well-matched cohort study, adds to the existing research by confirming the impaired humoral responses to COVID-19 mRNA vaccination in patients with IBD receiving anti-TNF-alpha immunosuppressive therapies. Furthermore, it identifies smoking as an independent risk factor for impaired vaccine immunogenicity in this group. Vaccines based on mRNA technology have been applied for the first time on a large scale against COVID-19. Due to their various advantages compared to conventional vaccines [38], we expect them to be used in the future against several other infections. Therefore, our findings may not be restricted to mRNA vaccines against SARS-CoV-2. Our findings sustain the implementation of individualized vaccine schedules in patients with different immunosuppressive therapies and encourage consideration of the smoking status. These findings also highlight the need for targeted interventions, such as smoking cessation programs and nutritional counseling, to optimize not only health but also vaccine responses in patients with IBD.

## Figures and Tables

**Table 1:** Characteristics of adult patients with IBD matched 1:3 to healthy HCW (HCW-healthy) and HCW with underlying diseases (HCW-plus) in a matched cohort design for age group and time since the second COVID-19 vaccination.

	IBD Patients ^1)^ (N = 110)	HCW-Healthy ^2)^ (N = 306)	*p*-Value ^4)^ HCW-Healthy vs. IBD pts.	HCW-Plus ^3)^ (N = 292)	*p*-Value ^5)^ HCW-Plus vs. IBD pts.
Gender—*females*	50 (45%)	203 (66%)	**<0.001**	238 (82%)	**<0.001**
Age (years), median (IQR)	44 (35–55)	42.5 (33–53)	0.342	44 (33–55)	0.622
Age groups			0.386		0.814
*18–30*	17 (15%)	51 (17%)		51 (17%)	
*31–40*	28 (25%)	84 (27%)		79 (27%)	
*41–50*	26 (24%)	78 (25%)		58 (20%)	
*51–60*	24 (22%)	71 (23%)		72 (25%)	
*>60*	15 (14%)	22 (7%)		32 (11%)	
BMI, median (IQR)	24.6 (21.5–28.1)	23.2 (21.0–25.7)	**0.013**	24.0 (21.3–26.9)	0.520
BMI categories ^6)^			**0.006**		0.878
*Underweight*	2 (2%)	7 (2%)		4 (1%)	
*Normal weight*	57 (52%)	203 (66%)		164 (56%)	
*Pre-obesity*	34 (31%)	78 (25%)		84 (29%)	
*Obesity all classes*	17 (15%)	18 (6%)		40 (14%)	
Clinical symptoms after the 1st vaccination	29 (26%)	88 (29%)	0.632	112 (38%)	**0.025**
Intensity of clinical symptoms after the 1st vaccination			0.197		0.057
*No symptoms*	81 (74%)	218 (71%)		180 (62%)	
*Mild or moderate symptoms*	26 (24%)	87 (28%)		106 (36%)	
*Severe symptoms*	2 (2%)	1 (0%)		6 (2%)	
Duration of clinical symptoms after the 1st vaccination			0.197		0.255
*Less than one week*	28 (100%)	83 (94%)		107 (96%)	
*More than one week*	0 (0%)	5 (6%)		5 (4%)	
Clinical symptoms after the 2nd vaccination	39 (35%)	149 (49%)	**0.017**	157 (54%)	**0.001**
Intensity of clinical symptoms after the 2nd vaccination			**0.044**		**0.003**
*No symptoms*	71 (65%)	157 (51%)		135 (46%)	
*Mild or moderate symptoms*	36 (33%)	131 (43%)		136 (47%)	
*Severe symptoms*	3 (3%)	18 (6%)		21 (7%)	
Duration of clinical symptoms after the 2nd vaccination			0.603		0.564
*Less than one week*	37 (95%)	144 (97%)		151 (97%)	
*More than one week*	2 (5%)	5 (3%)		5 (3%)	
Time difference to 2nd vaccination ^7)^			1.000		1.000
*>6 months*	7 (6%)	21 (7%)		21 (7%)	
*≤6 months*	103 (94%)	285 (93%)		271 (93%)	
Influenza vaccination during the last flu season 2020/2021	65 (63%)	135 (44%)	**<0.001**	150 (51%)	**0.040**
Any known allergy ^8)^	52 (48%)	110 (36%)	**0.031**	149 (51%)	0.554
Pollen allergy	35 (32%)	77 (25%)	0.161	104 (36%)	0.512
Drug allergy	23 (21%)	30 (10%)	**0.002**	42 (14%)	0.104
Current regular intake of vitamins, supplements, minerals, or fish oil	84 (78%)	80 (26%)	**<0.001**	129 (44%)	**<0.001**
Vitamin D supplements			**<0.001**		**<0.001**
*No vitamin D supplement*	29 (29%)	261 (85%)		212 (73%)	
*<1000 I.U. per day*	22 (22%)	23 (8%)		44 (15%)	
*≥1000 I.U. per day*	50 (50%)	22 (7%)		36 (12%)	
Smoking status			**0.044**		0.453
*Current smoker*	22 (20%)	38 (12%)		50 (17%)	
*Non-smoker/Previous smoker*	86 (80%)	268 (88%)		242 (83%)	

Results were presented in median and interquartile range (IQR) from 25% quartile to 75% quartile for continuous variables and in frequency (n) and column percentage (%) for categorical variables. ^1)^ IBD patient group includes patients with Crohn’s diseases or ulcerative colitis with an age of >=18 years under IBD specific drug therapy who received only mRNA vaccines for COVID-19 immunization. ^2)^ HCW-healthy include HCW with an age of >=18 years who did not report any underlying disease or any regular medication at enrollment and who received only mRNA vaccines for COVID-19 immunization. ^3)^ HCW-plus include HCW with an age of >=18 years who received only mRNA vaccines for COVID-19 immunization and reported at least one of the following underlying diseases: cardiovascular disease, chronic pulmonary disease, diabetes mellitus, thyroid dysfunction, hypothyroidism, chronic renal disease, renal insufficiency, chronic hepatic or gastrointestinal disease, chronic neurological disease or disorder, cancer, chronic haematological disease, or rheumatological disease; HCW-plus participants taking immunosuppressive/immunomodulatory drugs have been excluded from the analysis due to small number and heterogeneity of medication. ^4)^, ^5)^ *p*-values obtained by Mann–Whitney U-test for continuous variables, while Pearson’s chi-square test for categorical variables was used to determine the significant difference in the proportion of the respective factors between IBD patients and healthy HCW or IBD patients and HCW with underlying diseases, respectively. ^6)^ BMI categories were obtained by applying the WHO criteria available from https://iris.who.int/handle/10665/42330, accessed on 14 April 2024. ^7)^ Time difference to the second vaccination was calculated as the difference between the date of blood sampling at enrollment and the date of the second vaccination reported by participants in the initial questionnaire. ^8)^ Any known allergy reported in the questionnaire including drug allergy, food allergy, pollen allergy (allergic rhino-conjunctivitis), allergy against wasps, bee poison, contact allergy to chemicals, or anaphylaxis in the past. Significant *p*-values are given in bold. Abbreviations: BMI: body mass index, HCW: health care workers, IBD: inflammatory bowel disease, IQR: interquartile range.

**Table 2:** Factors associated with quantitative anti-SARS-CoV-2 spike Ig titers at baseline and at follow up in IBD patients ^1)^—results of multivariable generalized linear model (GLM), N = 96.

	At Baseline After 2nd Vaccination, N = 96				At Follow-Up After the 3rd Vaccination, N = 48			
	Anti-Spike Ig (BAU/mL), Median (IQR)	*p*-Value ^2)^	Multivariable GLM of Anti-Spike Ig (BAU/mL)		Anti-Spike Ig (BAU/mL), Median (IQR)	*p*-Value ^2)^	Multivariable GLM of Anti-Spike Ig (BAU/mL)	
			Coefficient (95% CI) ^3)^	*p*-Value ^4)^			Coefficient (95% CI) ^3)^	*p*-Value ^4)^
Age (years)	n.a.	n.a.	0.89 (0.77–1.03)	0.126	n.a.	n.a.	1.05 (0.83–1.31)	0.703
Gender								
*Females*	91 (16–902)	0.479	1		9838 (4312–21,285)	0.443	1	
*Males*	150 (46–798)		1.87 (1.22–2.87)	**0.004**	8457.5 (4142–18,340)		0.84 (0.52–1.37)	0.489
IBD type								
*Crohn’s disease*	91 (33–696)	0.168	1		6154 (2041–11,940)	**0.010**	1	
*Ulcerative colitis*	407 (58–914)		1.23 (0.80–1.89)	0.341	11,831 (8260–21,285)		2.19 (1.24–3.85)	**0.007**
IBD medications								
*Ustekinumab/Vedolizumab*	922 (456–2321)	**<0.001**	1		18,340 (8530–22,030)	**<0.001**	1	
*Anti-TNF* ^5)^	41 (14–90)		0.04 (0.03–0.06)	**<0.001**	4312 (2041–10,155)		0.40 (0.24–0.65)	**<0.001**
Chronic lung or pulmonary disease								
*No*	146 (42–914)	**0.032**	1		9330 (4142–18,535)	0.691	n.a.	n.a.
*Yes*	20 (1–249)		0.25 (0.11–0.57)	**0.001**	6225		n.a.	n.a.
Smoking status								
*Non-smoker/* *previous smoker*	172 (36–974)	0.154	1		9330 (3812–18,340)	0.701	1	
*Current smoker*	56 (33–453)		0.49 (0.29–0.84)	**0.010**	9020 (4814–18,945)		1.38 (0.76–2.50)	0.296
Time difference to 2. vaccination ^6)^								
*≤6 months*	122 (35–902)	0.735	1		9325 (4142–18,340)	0.181	1	
*>6 months*	286 (172–620)		0.50 (0.18–1.35)	0.171	24,798 (24,798–24,798)		0.62 (0.12–3.16)	0.563
Time difference to 3. vaccination (months) ^7)^	n.a.	n.a.	n.a.	n.a.	n.a.	n.a.	0.74 (0.52–1.05)	0.089

^1)^ IBD patients include patients with Crohn’s diseases or ulcerative colitis with an age of >=18 years under anti-TNF or ustekinumab/vedolizumab who received only mRNA vaccines for COVID-19 immunization with no missing data in covariates. ^2)^ *p*-value obtained from the Kruskal–Wallis test comparing the anti-Spike Ig (BAU/mL) in different strata among IBD patients. ^3)^ Results are given as coefficients of the model with their 95% confidence interval (CI) based on a GLM applying log nature transformation of anti-Spike IgG (BAU/mL) adjusted for gamma distribution due to the positive-skewed non-normal distribution of anti-Spike Ig value (BAU/mL). ^4)^ *p*-value obtained from Wald chi-square test in the final multivariable GLM determining the significance of the respective coefficient. Significant results were given in bold text. ^5)^ Anti-TNF drugs include infliximab, adalimumab or golimumab. ^6)^ Time difference to the second vaccination was calculated as the difference between date of blood sampling at enrollment and date of the second vaccination reported by participants in the initial questionnaire. This was categorized as ≤6 months and >6 months. ^7)^ Time difference to the third vaccination was calculated as the difference between date of blood sampling and date of the third vaccination reported by participants at follow-up and in the study mobile app. Significant *p*-values are given in bold. Abbreviations: IBD: inflammatory bowel disease, IQR: interquartile range, GLM: generalized linear model.

**Table 3:** Detection of neutralization antibody titers (NT) in adult patients with IBD categorized in non-neutralizing (NT ≥ 1:10 serum dilution) or neutralizing (NT < 1:10 serum dilution) depending on different variables after second COVID-19 vaccination.

	IBD Patients ^1)^ N = 110	Neutralizing Antibody Titer (NT) ^2)^		*p*-Value ^3)^
		Non-Neutralizing n = 69 (63%)	Neutralizing n = 41 (37%)	
Gender—females	50	31 (62%)	19 (38%)	0.885
Age (years), median (IQR)	44 (35, 55)	44 (36, 53)	44 (34, 57)	0.846
Age groups				0.557
*18–30*	17	9 (53%)	8 (47%)	
*31–40*	28	19 (68%)	9 (53%)	
*41–50*	26	19 (73%)	7 (27%)	
*51–60*	24	13 (54%)	11 (46%)	
*>60*	15	9 (60%)	6 (40%)	
BMI, median (IQR)	24.6 (21.5, 28.1)	24.5 (20.9, 27.7)	24.8 (22.1, 28.5)	0.477
BMI categories				0.980
*Underweight*	2	1 (50%)	1 (50%)	
*Normal weight*	57	36 (63%)	21 (37%)	
*Pre-obesity*	34	21 (62%)	13 (38%)	
*Obesity all classes*	17	11 (65%)	6 (35%)	
IBD type				0.927
*Crohn’s diseases*	65	41 (63%)	24 (37%)	
*Ulcerative colitis*	45	28 (62%)	17 (38%)	
IBD medications				**<0.001**
*Anti-TNF* ^4)^	53	44 (83%)	9 (17%)	
*Ustekinumab/Vedolizumab* ^5)^	44	16 (36%)	28 (67%)	
*Other medications* ^6)^	13	9 (69%)	4 (31%)	
Additional co-morbidity	84	52 (62%)	32 (38%)	0.748
Clinical symptoms after the 1st vaccination	29	20 (69%)	9 (31%)	0.418
Intensity of clinical symptoms after the 1st vaccination				0.679
*No symptoms*	81	49 (60%)	32 (40%)	
*Mild or moderate symptoms*	26	18 (69%)	8 (31%)	
*Severe symptoms*	2	1 (50%)	1 (50%)	
Clinical symptoms after the 2nd vaccination	39	25 (64%)	14 (36%)	0.825
Intensity of clinical symptoms after the 2nd vaccination				0.398
*No symptoms*	71	44 (62%)	27 (38%)	
*Mild or moderate symptoms*	36	22 (61%)	14 (39%)	
*Severe symptoms*	3	3 (100%)	0 (0%)	
Influenza vaccination during the last flu season 2020/2021	65	38 (58%)	27 (42%)	0.315
Any known allergy ^7)^	52	33 (63%)	19 (36%)	0.974
Food allergy	10	5 (50%)	5 (50%)	0.360
Pollen allergy	35	20 (57%)	15 (43%)	0.359
Drug allergy	23	14 (61%)	9 (39%)	0.785
Current regular intake of medication(s) beside IBD-related medications	79	50 (63%)	29 (37%)	0.925
Current regular intake of vitamin(s), supplement(s) mineral(s) or fish oil	84	51 (61%)	33 (39%)	0.365
Vitamin D supplements				0.161
*No Vitamin D supplementation*	29	22 (76%)	7 (24%)	
*<1000 I.U. per day*	22	11 (50%)	11 (50%)	
*≥1000 I.U. per day*	50	32 (64%)	18 (36%)	
Smoking status				**0.040**
*Current smoker*	22	18 (82%)	4 (18%)	
*Non-smoker/previous smoker*	86	50 (58%)	36 (42%)	

Results were presented in median and interquartile range (IQR) from the 25% quartile to the 75% quartile for continuous variables and in frequency (n) and row percentage (%) for neutralization antibody titer (NT) categories. ^1)^ IBD patients include patients with Crohn’s diseases or ulcerative colitis with an age of >=18 years under IBD specific drug therapy who received only mRNA vaccines for COVID-19 immunization. ^2)^ Neutralization antibody titers (NT) were categorized as non-neutralizing when NT ≥ 1:10 serum dilution and positive when NT < 1:10 serum dilution. Chi-square test was performed to compare the distribution of neutralization antibody titers in two categories, neutralizing and non-neutralizing NT. ^3)^ *p*-values obtained by Mann–Whitney U-test for continuous variables, while Pearson’s chi-square test for categorical variables was used to determine the significant difference in the proportion of the respective factors in IBD patients between non-neutralizing NT (≥1:10 serum dilution) and neutralizing NT (<1:10 serum dilution). *p*-values ≤ 0.05 were considered statistically significant. ^4)^ Anti-TNF antibody treatment with infliximab, adalimumab, and/or golimumab. ^5)^ Two patients received vedolizumab and ustekinumab treatment. ^6)^ Other medications including tofacitinib (n = 2), mesalazine (n = 4), immunomodulators (azathioprine, 6-mercaptopurin, methotrexate, or tacrolimus) (n = 5), and corticosteroids (n = 2). ^7)^ Any known allergy reported in the questionnaire including drug allergy, food allergy, pollen allergy (allergic rhino conjunctivitis), allergy against wasps, bee poison, contact allergy with chemicals, or anaphylaxis in the past. Significant *p*-values are given in bold. Abbreviations: IBD: inflammatory bowel disease, NT: neutralization antibody titers.

## Data Availability

Datasets can be made available in an irreversibly anonymized format upon reasonable request to the principal investigators.

## Supplementary Materials

The following supporting information can be downloaded at https://www.mdpi.com/article/10.3390/vaccines13070673/s1: Supplementary File S1: Flowchart for analysis of the IBD group of the RisCoin Study; Supplementary File S2: Stratification matching for IBD cohort; Supplementary File S3: Dietary habits and lifestyle factors among IBD patients, healthy HCW (HCW-healthy), and HCW with underlying diseases (HCW-plus); Supplementary File S4: Antibody neutralization titers (NT) after the second COVID-19 vaccination in adult patients with IBD categorized non-neutralizing (NT ≥ 1:10 serum dilution) or neutralizing (NT < 1:10 serum dilution) according to different dietary habits and lifestyle factors; Supplementary File S5: PSQ—Score in healthy HCW (HCW-healthy), HCW with underlying diseases (HCW-plus), and adult patients with IBD (A). Frequency of lower (≤33) or higher (>33) stress (B) and PSQ score based on the different determined domains (C).

## Abbreviations

The following abbreviations are used in this manuscript:

CI	confidence interval
FU	follow-up
GLM	Generalized Linear Model
HCW	healthcare workers
HCW-healthy	healthcare workers without comorbidities
HCW-plus	healthcare workers with medical conditions
IBD	inflammatory bowel disease
IQR	interquartile range
NT	neutralization antibody titers
PSQ	Perceived Stress Questionnaire
