# Supplementary material for: Factors Associated with Impaired Humoral Immune Response to mRNA Vaccines in Patients with Inflammatory Bowel Disease: A Matched-Cohort Analysis from the RisCoin Study
Source: Vaccines (Basel). 2025 Jun 23;13(7):673. doi: 10.3390/vaccines13070673 (PMC12299133; doi:10.3390/vaccines13070673)
Supplement: Supplementary file 1 [file vaccines-13-00673-s001.zip › vaccines-3610776-supplementary.pdf]

Factors associated with impaired humoral immune response to mRNA vaccines in patients with inflammatory bowel disease: a matched-cohort analysis from the RisCoin study

Katarina Csollarova[1+], Leandra Koletzko[2+], Thu Giang Le Thi[1,3], Paul R. Wratil[4,5], Ana Zhelyazkova[6] Simone Breiteneicher[2], Marcel Stern[4], Gaia Lupoli[4], Tobias Schwerd[1], Alexander Choukér[7], Veit Hornung[8], Oliver T. Keppler[4,5], Kristina Adorjan[9,10], Helga Paula Török[2*], Sibylle Koletzko[1,11*] on behalf of Members of RisCoin Study Group

[+]) shared first authors

[*]) shared last authors

Supplementary files

Supplementary File S1: Flowchart for analysis of the IBD group of the RisCoin Study................ 2
Supplementary File S2: Stratification matching for IBD cohort 3
Supplementary File S3: Dietary habits and lifestyle factors among IBD patients, healthy HCW (HCW-healthy) and HCW with underlying diseases (HCW-plus).. 6
Supplementary File S4: Antibody neutralization titers (NT) after the 2nd COVID19 vaccination in adult patients with IBD categorized non-neutralizing (NT ≥ 1:10 serum dilution) or neutralizing (NT < 1:10 serum dilution) according to different dietary habits and lifestyle factors................ 8
Supplementary File S5: PSQ – Score in healthy HCW (HCW-healthy), HCW with underlying diseases (HCW-plus) and adult patients with IBD (A). Frequency of lower (≤33) or higher (>33) stress (B) and PSQ-Score based on the different determined domains (C) 9

Supplementary File S1: Flowchart for analysis of the IBD group of the RisCoin Study

Data were extracted from RisCoin cohort with 4115 study participants (1).
Abbreviations: HCW: Health care workers, IBD: Inflammatory bowel disease, IQR: Inter-quartile range, FU1: First follow up after the 3rd COVID19-vaccination without any previous SARS-CoV 2 Infection.

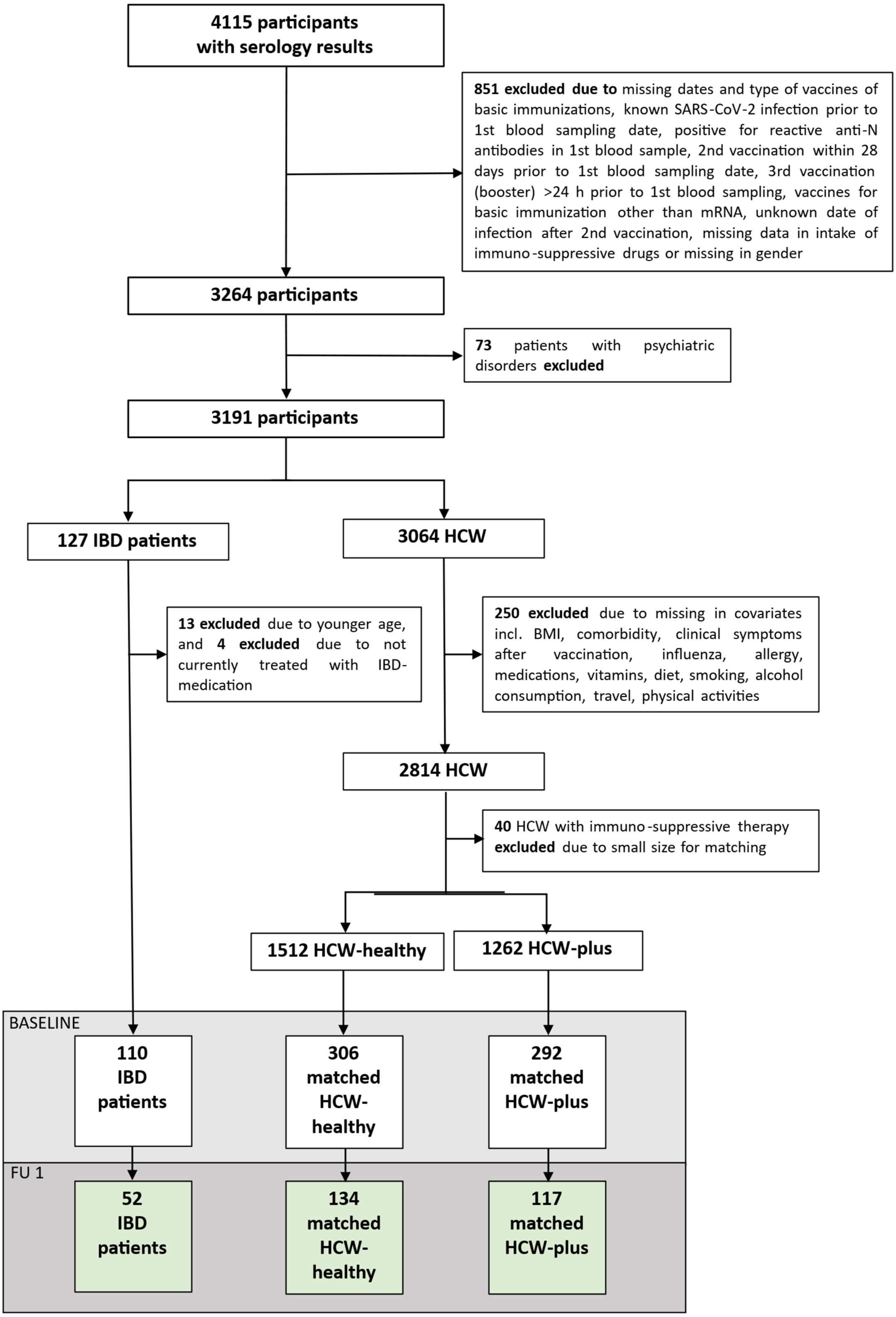

Supplementary File S2: Stratification matching for IBD cohort

For the immune response analysis, we excluded study participants with

- missing date and vaccine type of the 1st vaccination
- missing date and vaccine type of the 2nd vaccination
- known SARS-CoV-2 infection prior to sample date with or without date of confirmed PCR-test.
- reactive anti-N (nucleocapsid) antibodies detected at enrolment.
- the 3rd vaccination > 24 hours prior to sample collection at enrolment.
- the 2nd vaccination given within 28 days since sample collection date.
- vaccine other than mRNA + mRNA

We excluded study participants with missing data for the severity of clinical symptoms following the first and second vaccinations, as well as for influenza vaccination, allergy, consumption of mineral supplement(s), vitamin D, fish oil, vegetables and fruit, smoking status (including tobacco products, e-cigarettes, hookah pipes), alcohol consumption, travel abroad in the past twelve months, and physical activity. Furthermore, we excluded patients with psychiatric disorders and patients with IBD under the age of 18 years and those not currently receiving IBD-related treatments. Following this data selection process, 110 patients with IBD and 2814 HCW were included (Supplementary file 1).

At the data exploration step, our findings indicate that, immune response quantified by anti-spike Ig (BAU/ml) was lower in HCW reported with chronic diseases and those without. In addition, immune response altered with older ages. Of note, participants with chronic diseases are more likely older than those without. From these reasons, we stratified healthcare workers (HCWs) as either healthy (HCW-healthy) or HCW with underlying diseases (HCW-plus). Healthy HCWs were defined as those who did not report any underlying disease or regular medication at the time of enrolment. HCW-plus included all HCWs who reported regular medication or had at least one of the following underlying diseases: cardiovascular disease, chronic pulmonary disease, diabetes mellitus, thyroid dysfunction, hypothyroidism, chronic renal disease, renal insufficiency, chronic hepatic or gastrointestinal disease, chronic neurological disease or disorder, cancer, transplantation, chronic hematological disease, rheumatological disease, or primary immunodeficiency disorders. HCW currently treated with immunosuppressive drugs were separated and not included into the HCW-plus group due to the limited number of participants and the heterogeneity regarding immunosuppressive treatment in this subgroup. After stratified the HCW cohort, we identified 1512 HCW-healthy (median age: 35 years) und 1262 HCW-plus (median age: 44 years), $p<0.001$. The level of anti-spike Ig (BAU/ml) of HCW-healthy were significantly higher than of HCW-plus, median value: 1044 BAU/ml (IQR: 581-1799) vs 961 BAU/ml (IQR: 514-1631), $p=0.001$. The stratification of HCW allowed us to compare the immune response of patients with IBD with HCW, who also had chronic diseases but were not treated with immunosuppressive mediations.

Moreover, both the levels of quantitative anti-spike Ig (BAU/ml) and neutralization antibody titers decline with time since the last vaccination to the blood sample date increases. This following table shows the significant difference of levels of quantitative

anti-spike Ig (BAU/ml) and neutralization antibody titers in HCW-healthy and HCW-plus who had been vaccinated ≤6 months vs >6 months prior to sample date.

	**Total**	**Time difference prior to sample date**		**p-value**
		**≤ 6 months**	**> 6 months**	
**Anti-spike Ig (BAU/ml), median (IQR)**				
HCW-healthy (N=1512)	1044 (581-1799)	1674 (1050-2623), N=553	789 (459-1301), N=959	**<0.001**
HCW-plus (N=1262)	961 (514-1631)	1548 (946-2223), N=355	788 (441-1305), N=907	**<0.001**
**Neutralization antibody titers (1:10), median (IQR)**				
HCW-healthy (N=1512)	53 (10-156)	107 (30-293), N=553	33 (10-122), N=959	**<0.001**
HCW-plus (N=1262)	51 (10-152)	93 (27-186), N=355	39 (10-136), N=907	**<0.001**

In addition, according to Germany's national vaccination strategies in 2021/2022, HCW were given priority for vaccination, whereas patients with IBD were vaccinated approximately 2-3 months later. In the RisCoin study, 63% of HCW-plus (N=1262) and 72% of HCW-healthy (N=1512) had received their vaccinations >6 months prior to the blood sample date, compared to just 6% of IBD patients who were vaccinated >6 months prior to the blood sample date.

Our aim is to study the immune response in patients with IBD after vaccination by comparing it to HCW who may experience similar environmental or infectious exposures as patients but are generally healthier. This comparison allows us to precisely identify factors specifically associated with disease-related immune alterations. To ensure valid comparisons with HCW cohorts, it is essential to have groups that are comparable in age and the timing of vaccination. Consequently, we applied stratification matching approach to mitigate the confounding effect of age and vaccination timing on the immune response. We created 10 respective strata based on 5 age groups (18-30, 31-40, 41-50, 51-60, >60 years) and 2 groups of early or later vaccination (time between the sample collection date and the 2nd vaccination >6 months and ≤6 months). We then randomly selected HCWs within each stratum, with a ratio of 1 IBD patient to 3 HCWs, from HCW-healthy or HCW-plus, respectively. In the strata, where the number of HCWs is greater than the required number of HCWs (1:3), we selected HCWs randomly applying SAS procedure, proc select, with defined seed number. In the strata, where we could not find the required number of matched HCWs, we selected all HCWs given in this stratum.

Applying stratification matching, we selected 306 healthy HCW and 292 HCW with underlying diseases as controls of the IBD cohort for further analysis (**Supplementary file 1**). This approach allows us to ensure that HCWs within each stratum are comparable with IBD patients regarding age and time between sample collection date and the 2nd vaccination.

Stratification matching healthy HCW to IBD patients						
	Before matching			After matching		
Factor, n (%, row percentage)	HCW-healthy (N=1512)	IBD patients (N=110)	P-value	HCW-healthy (N=306)	IBD patients (N=110)	P-value
**Females (%)**	1026 (68%)	50 (45%)	**<0.001**	203 (66%)	50 (45%)	**<0.001**
**Age (years)**	35 (28-47)	44 (35-55)	**<0.001**	43 (33-53)	44 (35-55)	0.342
**Age groups**			**<0.001**			0.386
18-30	503 (33%)	17 (15%)		51 (17%)	17 (15%)	
31-40	440 (29%)	28 (25%)		84 (27%)	28 (25%)	
41-50	276 (18%)	26 (24%)		78 (25%)	26 (24%)	
51-60	243 (16%)	24 (22%)		71 (23%)	24 (22%)	
>60	50 (3%)	15 (14%)		22 (7%)	15 (14%)	
**Time difference to 2nd vaccination**			**<0.001**			1.000
≤ 6 months	553 (37%)	103 (94%)		285 (93%)	103 (94%)	
> 6 months	959 (63%)	7 (6%)		21 (7%)	7 (6%)	
**Stratification matching HCW with underlying diseases to IBD patients**						
	Before matching			After matching		
Factor, n (%, row percentage)	HCW-plus (N=1262)	IBD patients (N=110)	P-value	HCW plus (N=292)	IBD patients (N=110)	P-value
**Females (%)**	1056 (84%)	50 (45%)	**<0.001**	238 (82%)	50 (45%)	**<0.001**
**Age (years)**	43 (31-55)	44 (35-55)	**<0.001**	44 (33-55)	44 (35-55)	0.622
**Age groups**			0.176			0.814
18-30	294 (23%)	17 (15%)		51 (17%)	17 (15%)	
31-40	276 (22%)	28 (25%)		79 (27%)	28 (25%)	
41-50	225 (18%)	26 (24%)		58 (20%)	26 (24%)	
51-60	325 (26%)	24 (22%)		72 (25%)	24 (22%)	
>60	142 (11%)	15 (14%)		32 (11%)	15 (14%)	
**Time difference to 2nd vaccination**			**<0.001**			1.000
≤ 6 months	355 (28%)	103 (94%)		271 (93%)	103 (94%)	
> 6 months	907 (72%)	7 (6%)		21 (7%)	7 (6%)	

Results were presented in frequency (n) and column percentage (%). P-values obtained by Pearson's Chi-square test to determine the significant difference in the proportion of the respective factors between IBD patients and healthy HCW (HCW-healthy) or IBD patients and HCW with underlying diseases (HCW-plus), respectively.

Abbreviations: HCW: Health care workers, IBD: Inflammatory bowel disease, IQR: Inter-quartile range.

Supplementary File S3: Dietary habits and lifestyle factors among IBD patients, healthy HCW (HCW-healthy) and HCW with underlying diseases (HCW-plus)

Factor n (%, column percent)	IBD patients[1)] (N=110)	HCW-healthy[2)] (N=306)	P-Value[4)] HCW-healthy vs. IBD patients	HCW-plus[3)] (N=292)	P-Value[5)] HCW-plus vs. IBD patients
**Alcohol consumption**	62 (57%)	230 (75%)	**0.001**	207 (71%)	**0.014**
**How often do you drink alcohol?**			**0.003**		**0.038**
No alcohol consumption	46 (43%)	76 (25%)		85 (29%)	
Once a month or less	9 (8%)	22 (7%)		20 (7%)	
2-4 times per month	27 (25%)	108 (35%)		98 (34%)	
≥ 2 times a week	24 (23%)	100 (33%)		89 (30%)	
**Dietary habits**					
Regular consumption of vegetables and fruits, moderate portions of meat or meat products	56 (51%)	178 (58%)	0.188	168 (58%)	0.233
Almost daily consumption of meat and meat products	27 (25%)	49 (16%)	**0.047**	45 (15%)	**0.033**
Frequent or regular consumption of ready-to-eat meals or food	9 (8%)	19 (6%)	0.479	20 (7%)	0.645
Regular fish consumption	25 (23%)	47 (15%)	0.080	46 (16%)	0.102
**Diet**					
Pescatarian diet	6 (5%)	31 (10%)	0.139	32 (11%)	0.093
Vegan (solely plant-based diet)	3 (3%)	7 (2%)	0.796	10 (3%)	0.725
Weight control diet	1 (1%)	6 (2%)	0.462	10 (3%)	0.168
Avoiding special foods i.e. due to food allergy or intolerance	22 (20%)	11 (4%)	**<0.001**	24 (8%)	**<0.001**
Others	5 (5%)	19 (6%)	0.521	15 (5%)	0.808
**Number of portions of seafood or fish consumed in the past 7 days**			0.478		0.078
0	38 (35%)	133 (43%)		135 (46%)	
1-2	67 (61%)	164 (54%)		148 (51%)	
3-4	3 (3%)	7 (2%)		9 (3%)	
≥ 5	1 (1%)	2 (1%)		0 (0%)	
**Number of portions of vegetables and fruits consumed on average per day**			**0.003**		**0.042**
0	7 (6%)	12 (4%)		14 (5%)	
1-2	82 (75%)	178 (58%)		183 (63%)	
3-4	18 (17%)	105 (34%)		80 (27%)	
≥ 5	2 (2%)	11 (4%)		15 (5%)	
**Having travelled abroad in the last 12 months**			**0.006**		0.095
Never	41 (38%)	68 (22%)		78 (27%)	
1-3 times	57 (52%)	208 (68%)		184 (63%)	
≥ 4 times	11 (10%)	30 (10%)		30 (10%)	
**Having moderate to heavy physical activity**			0.164		0.756
None	27 (25%)	51 (17%)		63 (22%)	
Once per week	31 (28%)	90 (29%)		91 (31%)	
More than once per week	51 (47%)	165 (54%)		138 (47%)	

Results are presented in median and interquartile range (IQR) from the 25% to the 75% quartile for continuous variables and in frequency (n) and column percentage (%) for categorical variables.

[1)] IBD patients group include IBD patients in drug therapy for their bowel disease with an age of >18 years who received only mRNA vaccines for COVID19 immunization

[2)] HCW-healthy include all HCW who did not report any underlying disease or any medication intake at enrolment.

[3)] HCW-plus include all HCW with an age of >18 years who received only mRNA vaccines for COVID19 immunization only mRNA vaccines and reported regular medication, but no immunosuppressive drugs, or at least one of the following underlying diseases: cardiovascular disease, chronic pulmonary disease, diabetes mellitus, thyroid dysfunction, hypothyroidism, chronic renal disease, renal insufficiency, chronic hepatic or gastrointestinal disease, chronic neurological disease or disorder, cancer, transplantation, chronic hematological disease, rheumatological disease, or primary immunodeficiency disorder. HCW-plus participants taking immunosuppressive/immunomodulatory drugs have been excluded from the analysis due to small number and heterogeneity of medication.[4)] [5)] P-values obtained by Mann–Whitney-U-test for continuous variables, while Pearson's Chi-square test for categorical variable to determine the significant difference in the proportion of the respective factors between IBD patients and healthy HCW or IBD patients and HCW with underlying diseases, respectively.

Abbreviations: HCW: Health care workers, IBD: Inflammatory bowel disease, IQR: Inter-quartile range.

Supplementary File S4: Antibody neutralization titers (NT) after the 2nd COVID19 vaccination in adult patients with IBD categorized non-neutralizing (NT ≥ 1:10 serum dilution) or neutralizing (NT < 1:10 serum dilution) according to different dietary habits and lifestyle factors

Factor n (%, row percentage)	IBD patients[1)] (N=110)	Neutralization antibody titers (NT)[2)]		P-value[3)]
		non-neutralizing n=69 (63%)	neutralizing n=41 (37%)	
**Current alcohol consumption**	62	42 (68%)	20 (32%)	0.407
**Current regular intake of vitamins, supplements, minerals, or fish oil**				
Fish oil	4	2 (50%)	2 (50%)	0.584
Mineral supplement(s)	8	6 (75%)	2 (25%)	0.464
**Dietary habits**				
Regular consumption of vegetables and fruits, moderate portions of meat or meat products	56	31 (55%)	25 (45%)	0.104
Almost daily consumption of meat and meat products	27	19 (70%)	8 (30%)	0.344
Frequent or regular consumption of ready-to-eat meals or food	9	6 (67%)	3 (33%)	0.799

Results were presented in median and interquartile range (IQR) from 25% quartile to 75% quartile for continuous variables and in frequency (n) and row percentage (%) for NT.

[1)] IBD patients include patients with Crohn´s diseases or ulcerative colitis with an age of >18 years under IBD specific drug therapy who received only mRNA vaccines for COVID19 immunization.

[2)] Neutralization antibody titers (NT) was categorized as non-neutralizing when NT ≥ 1:10 (serum dilution) and neutralizing when NT < 1:10 (serum dilution). Chi-square test was performed to compare the distribution of neutralization antibody titers in two categories, neutralizing and non-neutralizing NT.

[3)] P-values obtained by Mann–Whitney-U-test for continuous variables, while Pearson’s Chi-square test for categorical variable to determine the significant difference in the proportion of the respective factors in IBD patient between Non-neutralizers (NT ≥ 1:10 serum dilution) and Neutralizer (NT < 1:10 serum dilution)

. P-values ≤ 0.05 were considered statistically significant.

Abbreviations: IBD: Inflammatory bowel disease, NT: Neutralization antibody titers

Supplementary File S5: PSQ – Score in healthy HCW (HCW-healthy), HCW with underlying diseases (HCW-plus) and adult patients with IBD (A). Frequency of lower (≤33) or higher (>33) stress (B) and PSQ-Score based on the different determined domains (C)

A)

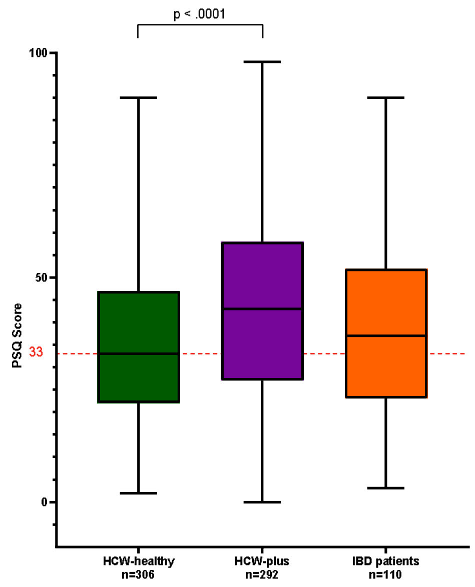

B)

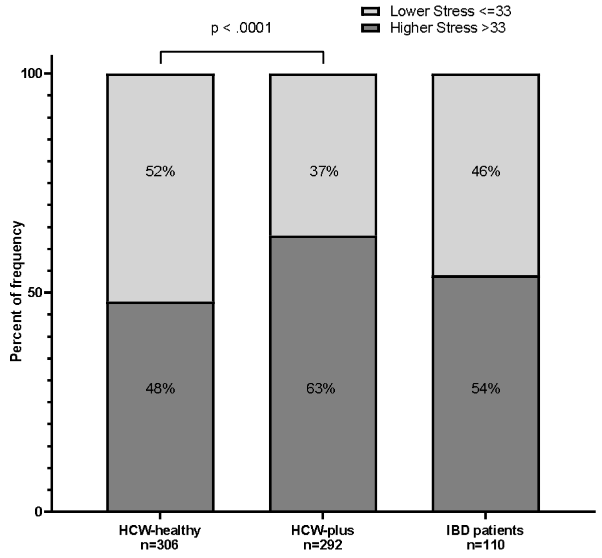

C)

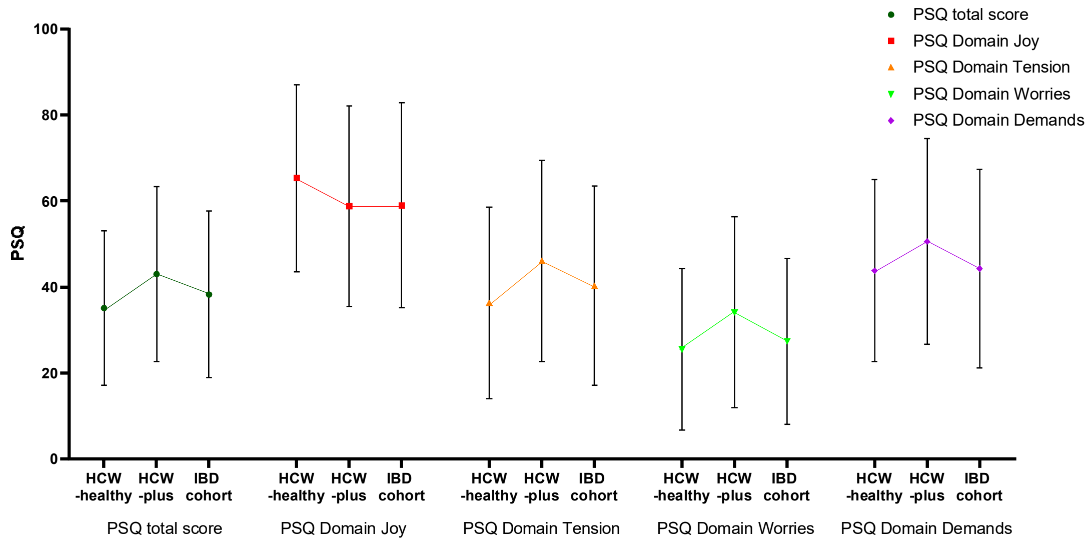

Comparison of Perceived Stress Questionnaire - Score (PSQ-Score) between IBD patients, healthy HCW (HCW-healthy) and HCW with underlying diseases (HCW-plus) quantitative (A) and in proportion to their respective cohort (%) (B). Comparison in PSQ scores in different stress domains (C). We applied the mean PSQ score of the healthy cohort presented in Fliege et al 2005 (2) as reference score to define average higher stress (PSQ > 33) and average lower stress (PSQ ≤ 33).

Abbreviations: IBD: Inflammatory bowel disease, HCW: Health care worker, PSQ: Perceived Stress Questionnaire

References

1. Koletzko S, Le Thi TG, Zhelyazkova A, Osterman A, Wichert SP, Breiteneicher S, et al. A prospective longitudinal cohort study on risk factors for COVID-19 vaccination failure (RisCoin): methods, procedures and characterization of the cohort. Clin Exp Med. 2023;8: 4901-4917

2. Fliege H, Rose M, Arck P, Walter OB, Kocalevent RD, Weber C, Klapp BF. The Perceived Stress Questionnaire (PSQ) reconsidered: validation and reference values from different clinical and healthy adult samples. Psychosom Med. 2005;67(1):78-88.
